# Synaptic Remodeling in the Dentate Gyrus, CA3, CA1, Subiculum, and Entorhinal Cortex of Mice: Effects of Deprived Rearing and Voluntary Running

**DOI:** 10.1155/2010/870573

**Published:** 2010-05-25

**Authors:** Andrea T. U. Schaefers, Keren Grafen, Gertraud Teuchert-Noodt, York Winter

**Affiliations:** ^1^Department of Biology, Bielefeld University, Universitätsstraße 25, 33615 Bielefeld, Germany; ^2^Department of Biology, Humboldt University, Berlin, Germany; ^3^Cluster of Excellence NeuroCure, Charité-Universitätsmedizin Berlin, Berlin, Germany

## Abstract

Hippocampal cell proliferation is strongly increased and synaptic turnover decreased after rearing under social and physical deprivation in gerbils (*Meriones unguiculatus*). We examined if a similar epigenetic effect of rearing environment on adult neuroplastic responses can be found in mice (*Mus musculus*). We examined synaptic turnover rates in the dentate gyrus, CA3, CA1, subiculum, and entorhinal cortex. No direct effects of deprived rearing on rates of synaptic turnover were found in any of the studied regions. However, adult wheel running had the effect of leveling layer-specific differences in synaptic remodeling in the dentate gyrus, CA3, and CA1, but not in the entorhinal cortex and subiculum of animals of both rearing treatments. Epigenetic effects during juvenile development affected adult neural plasticity in mice, but seemed to be less pronounced than in gerbils.

## 1. Introduction

Synaptic plasticity in higher brain regions of mammals is not restricted to prenatal ontogenesis, but occurs also postnatally during development and even in the adult brain. Besides *reactive synaptogenesis* following lesions and deafferentation [1–3], synaptic turnover in axon terminals is a *naturally occurring process* of structural plasticity in the adult rodent brain [4–7]. These nondegenerative synaptic degradation events of remodeling synaptic connections or revision of transmitter release [8, 9] are activity-dependent processes [6, 10–16] permitting the animal to learn and to adapt to environmental changes ([17], reviewed in [18]).

Within the hippocampal formation, the generation of new neurons (neurogenesis) adds a further level of complexity of structural plasticity to the preexisting neuronal network, even in the mature brain (reviewed in [9, 19, 20]). There are strong indications that neurogenesis is interrelated to synaptic remodeling, since new neurons demand synaptic integration (reviewed in [9, 19]) and neuron survival depends on functional integration into the existing neuronal network [21–23]. Both processes, neurogenesis and synaptic remodeling, are susceptible to the type and degree of animal-environment interactions [5, 24–29] and voluntary physical activity [30–34]. In gerbils, rates of synaptic remodeling in the inner molecular layer of the dentate gyrus are lower when adult hippocampal cell proliferation is increased. This can be observed in individuals reared under social and physical deprivation (impoverishment), which have higher rates of adult hippocampal cell proliferation than enriched-reared animals, while synaptic turnover rates in the inner molecular layer are reduced ([5, 35], see also [16] for a theoretical model). First observations in CD1 mice suggest that rates of cell proliferation and cell survival are not affected by environmental conditions during rearing per se. However, only deprived-reared animals responded to wheel running with a significant increase in neurogenesis suggesting a different neuroplastic development (in prep.). The present study was conducted to find out to which extent the findings on the epigenetic effects of juvenile environmental stimulation or deprivation on synaptic plasticity in gerbils are also valid for the mouse model. Specifically, we examined whether CD1 mice reared under different environmental conditions have different rates of synaptic turnover in adulthood. Thereby, we tested the prediction of an inverse relationship between adult hippocampal cell proliferation and synaptic turnover in the inner molecular layer as suggested by the model of Teuchert-Noodt and coworkers [5, 9, 16]. To obtain a comprehensive picture of the effects of environmental conditions during rearing on synaptic turnover in the regions down- and upstream of the neurogenesis site in the dentate gyrus, we included the CA3 and CA1 regions of the hippocampus proper and the subiculum and entorhinal cortex in our analyses. We applied the same rearing-condition treatment as in previous gerbil studies, which, in contrast to most studies in mice and rats, excluded running wheels from the enriched environment during rearing. We scored synaptic turnover by quantifying lysosomal accumulations in degrading axon terminals after Gallyas silver impregnation [36].

## 2. Material and Methods

Presynaptic degradation is a prominent form of synaptic remodeling. The *nondegenerative processes* are characterized by a transient accumulation of secondary lysosomes and degenerating organelles in the degrading axonal terminal [8, 37, 38]. While primary lysosomes are ubiquitous in the cell, the accumulation of secondary lysosomes is a prominent feature of turnover processes [37]. Electron microscopy and ultrathin section analyses of silver-impregnated brain sections have convincingly shown that the Gallyas silver impregnation selectively stains secondary lysosomes, degrading mitochondria, lamellar bodies, and multivesicular bodies in remodeling axonal terminals [8, 36, 38, 39]. It is still the only method available to specifically detect presynaptic remodeling processes by staining the transient phase of lysosomal accumulations. 

### 2.1. Animals and Rearing Conditions

**Figure 1:**
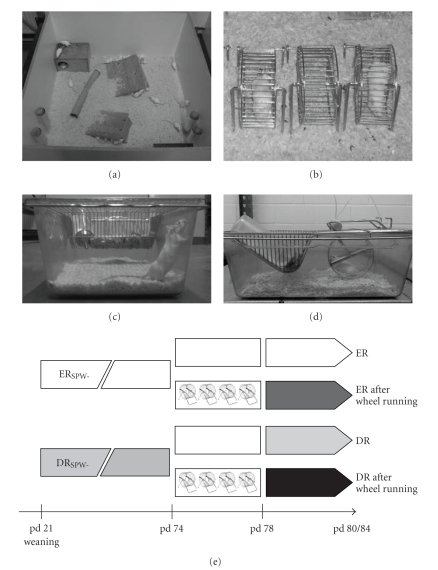
(a) Mice in socially and physically enriched-rearing condition. (b) Wheel running of group-housed individuals; (c) Mouse in a cage of the socially and physically deprived-rearing condition. (d) Mouse wheel running in its individual cage as in (c). (e) Experimental design with time axis showing animal age in postnatal days (pd); pd 21–74, rearing phase under socially and physically enriched conditions (ERs) or socially and physically deprived conditions (DRs); pd 74–78, 4-day wheel running challenge for the half of each rearing group; synaptic-remodeling rates were assessed between pd 80 and 84.

CD1 mice were bred in our facilities in standard type III cages (42 × 26 × 18 cm) with one litter per cage. At weaning (postnatal day (pd) 21), 25 male animals were transferred to one of the two rearing conditions, enriched (ER_SPW-_) or socially (_S_) and physically (_P_) deprived (DR_SPW-_) rearing, both without running wheels (_W-_). Rearing conditions were defined relative to three parameters: the social environment (single- or group-housed) denoted by _S_, the physical environment (small or large space and presence or absence of environmental objects such as tunnels, tubes, and solid hiding places) denoted by _P_, and the absence of a running wheel under all conditions denoted by _W-_. Accordingly, ER_SPW-_ animals (*n* = 13) were kept in one group in a large enclosure (200 × 100 × 50 cm) that, in addition to animal bedding, contained items such as tunnels, tubes, and solid hiding places (Figure 1(a)). DR_SPW-_ animals (*n* = 12) were reared individually in standard type III cages that contained no other materials than animal bedding and two sheets of toilet paper (Figure 1(c)). All animals were on a 12-h-light-dark schedule and received laboratory animal feed (Höveler, Dormagen, Germany) and water *ad libitum*. Animals were kept under their respective rearing conditions until pd 74 (Figure 1(e)).

### 2.2. Wheel Running Challenge

On pd 74, the enrichment (tunnels, toys, etc.) was removed from the enclosure of the ER animals and the enclosure was divided into two equal sections by a wooden plate. In one section, 6 animals of the group were given running wheels (one per animal; Figure 1(b)). Accordingly, 5 DR animals received a running wheel in their individual cages (Figure 1(d)). The wheel running challenge lasted 4 days from pd 74 to pd 78. After the wheel running challenge the animals remained under their respective conditions without running wheels for 3–7 days (Figure 1(e)).

### 2.3. Tissue Preparation and Staining

Animals were deeply anesthetized by an overdose of diethyl ether and transcardially perfused with 5% formalin. Brains were dissected and subsequently kept in formalin for at least two weeks at 4°C. The left hemispheres were freeze-sectioned into 60 *μ*m thick coronarl sections and collected in distilled water. Every second section from the septal pole to the temporal pole of the hippocampus was used for staining. In modification of the procedure originally described by Gallyas et al. [36], the free-floating sections were prepared in an alkalinic solution (pH 13) containing 9% sodium hydroxide and 1% ammonium nitrate and subsequently silver-impregnated by a silver nitrate solution containing 9% sodium hydroxide, 16% ammonium nitrate, and approximately 50% silver nitrate. The optimum silver concentration was determined by examining stained test sections by light microscopy. After impregnation, the sections were rinsed three times in changing rinsing solutions (solution: 30% ethyl-alcohol with 0.5 g sodium carbonate mixed with 1% ammonium nitrate). The developer contained 15 mL 40% formalin and 0.5% citric acid in 1000 mL 10% ethyl alcohol. After another rinsing step, the sections were air-dried, mounted on slides, and embedded in DePeX (Serva, Heidelberg, Germany).

### 2.4. Measuring Fields and Computerized Assessment of Lysosomal Accumulations

**Figure 2:**
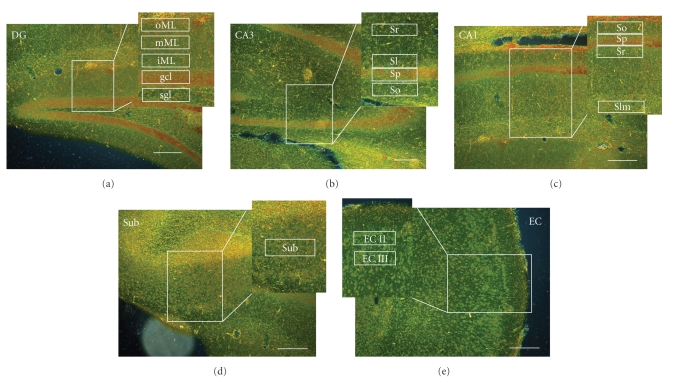
Dark field light microscopy images of the dentate gyrus (DG), CA3, CA1, subiculum (Sub), and entorhinal cortex (EC) with silver-impregnated lysosomal accumulations appearing as bright precipitates (Gallyas technique). In the dentate gyrus, five measuring fields were positioned in the outer (oML), intermediate (mML), and inner molecular layer (iML), as well as in the granule cell (gcl) and subgranular layer of both the suprapyramidal and infrapyramidal blades (not indicated). In CA3, measuring fields were positioned in the stratum radiatum (Sr), stratum lucidum (Sl), stratum pyramidale (Sp), and stratum oriens (So). In CA1, an additional field was set in the stratum lacunosum-moleculare (Slm). One measuring field was positioned in the subiculum and two in the entorhinal cortex in lamina II (EC II) and III (EC III). Scale bar = 200 *μ*m.

Silver-impregnated lysosomal accumulations can be viewed and counted in the dark field at 100-fold magnification. For computer-assisted quantification of precipitates, pictures were taken of the dentate gyrus, CA1, CA3, subiculum, and entorhinal cortex using a light microscope (Olympus BX61 with a UplanFl 10 ×/0.30 objective, Olympus Europa Holding GmbH, Hamburg, Germany), a digital camera (Color View, Soft Imaging Systems GmbH, Münster, Germany), and software for image analysis (Cell*, Olympus Soft Imaging Solutions, Münster, Germany). For the subiculum and entorhinal cortex, 6–8 sections per animal, and for the dentate gyrus, CA1, and CA3, 14–20 sections per animal were used for quantification. Within the respective areas, predefined measuring fields were set to estimate the number of precipitates per layer. Measuring fields consisted of three joined rectangular-shaped subfields (200 × 50 pixels) sized 600 × 50 pixels in total, which covered almost the entire suprapyramidal and infrapyramidal blade of the dentate gyrus. Their height was fitted to the granule cell layer. In every measuring field, precipitates were counted by a self-developed classification algorithm implemented in MATLAB Vers. 6 [16]. This algorithm consists of four steps. first, the digital image of one measuring field is transferred to an intensity gray scale image. Secondly, the image is normalized by setting its median to zero level. Then, a linear filter is applied to the image (for matrix see [16]), enhancing the contrast between a few-pixels sized, bright silver precipitates and the dark background. A threshold allows the distinction of precipitates and background. Last, white spots of detected precipitates were thinned out to a single pixel to count their number irrespective of their size. These four steps were repeated for all measuring fields to assess the average number of precipitates in every measured layer [16]. Within the dentate gyrus, measuring fields were set in the outer, intermediate, and inner molecular layer, in the granule cell layer and subgranular layer. Within the CA3 region, fields were set in the stratum oriens, stratum pyramidale, stratum lucidum, and stratum radiatum. Within the CA1 region, measuring fields were set in the stratum oriens, stratum pyramidale, stratum radiatum, and stratum lacunosum-moleculare. Within the subiculum, fields were set in layer III, within the entorhinal cortex in layers II and III. Microscopy images of the different hippocampal regions and positions of the measuring fields are presented in Figure 2. Pictures and measurements were performed by a person blind to the group membership of the individuals.

### 2.5. Statistical Analysis

Because of the high connectivity of the layers within the different hippocampal regions, statistical analyses were conducted for each of the different regions, comprising all layers, respectively. Thus, factorial analyses of variance (ANOVA) with the dependent variable “precipitates” and the categorical predictors “rearing condition”, “wheel running”, and “layer” were performed in all examined regions separately. Subsequent specific comparisons were made with Unequal N HSD post hoc test. All statistical analyses were computed with Statistica 6 (StatSoft, Tulsa, USA).

## 3. Results

**Figure 3:**
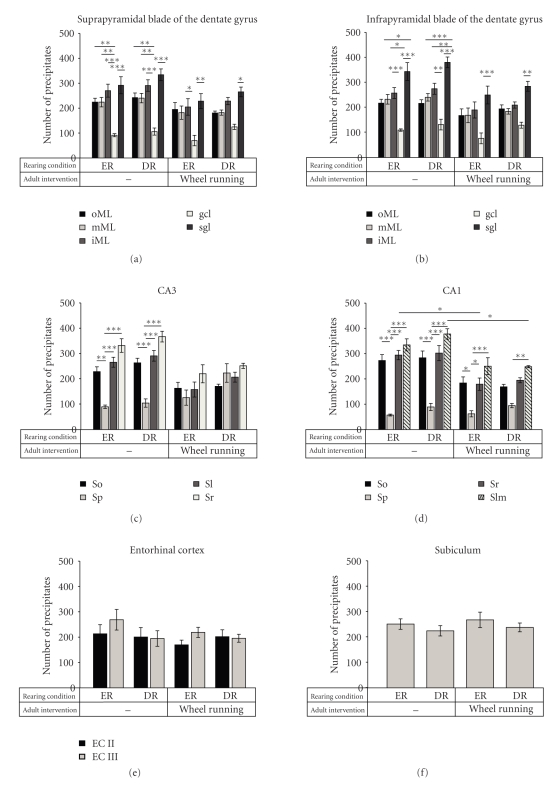
Number of precipitates for each layer in the (a) suprapyramidal and (b) infrapyramidal blade of the dentate gyrus, in the (c) CA3, (d) CA1, (e) entorhinal cortex, and (f) subiculum of the different treatment groups; ER: socially and physically enriched-reared animals; DR: socially and physically deprived-reared animals; wheel running: additional wheel running challenge in adulthood (pd 74–78); oML: outer molecular layer; mML: intermediate molecular layer; iML: inner molecular layer; gcl: granule cell layer; sgl: subgranular layer; spb: suprapyramidal blade; ipb: infrapyramidal blade; So: stratum oriens, Sp: stratum pyramidale; Sl: stratum lucidum; Sr: stratum radiatum; Slm: stratum lacunosum-moleculare; EC II: entorhinal cortex lamina II; EC III: entorhinal cortex lamina III; data given as means ± S.E.M., asterisks denote significant differences: *P < .05, **P < .01, ***P < .001.

The amount of synaptic turnover in axon terminals was assessed by quantification of silver-impregnated lysosomal accumulations in the dark field (Figure 3). The data given below are the mean total number of precipitates in the measuring fields of each layer.

### 3.1. Layer-Specific Differences in the Number of Lysosomal Accumulations in the Dentate Gyrus and Hippocampus Proper

In our socially and physically enriched-reared control animals significant differences appeared in the number of precipitates between the layers of the dentate gyrus (ANOVA, layer *F*_9,21_ = 35.4, *P* < .0001, Figures 3(a) and 3(b)), CA3 (ANOVA, layer *F*_3,84_ = 31.9, *P* < .0001, Figure 3(c)), and CA1 (ANOVA, layer *F*_3,84_ = 82.3, *P* < .0001, Figure 3(d)). 

In the dentate gyrus, these differences in the number of precipitates were mainly present between the granule cell layer, with a rather low density (92 in the suprapyramidal and 109 in the infrapyramidal blade), and the molecular layers (Post hoc Unequal N HSD, *P* < .01–.001) as well as the subgranular layer (Post hoc Unequal N HSD, *P* < .001), while the subgranular layer showed the highest number of precipitates (292 in the suprapyramidal and 344 in the infrapyramidal blade) followed by the inner molecular layer (270 in the suprapyramidal and 258 in the infrapyramidal blade, Figures 3(a) and 3(b)).

In CA3, a similar pattern could be observed, with most significant differences between the rather acellular layers and the pyramidal cell layer with the lowest number of precipitates (91; Post hoc Unequal N HSD, *P* < .001). However, there was also a trend towards a difference between the stratum radiatum, with the highest number of precipitates (333), and the stratum oriens (230; Post hoc Unequal N HSD, *P* = .07, Figure 3(c)).

CA1 resembled the distribution of CA3, with the largest differences between the rather acellular layers and the pyramidal cell layer with the lowest amount of precipitates (57; Post hoc Unequal N HSD, *P* < .001). However, in CA1, the stratum lacunosum-moleculare appeared with the highest number of precipitates (335) although the difference from the stratum oriens or stratum radiatum was not significant (Figure 3(d)).

### 3.2. No Differences in the Distribution of Precipitates between Socially and Physically Enriched- and Deprived-Reared Animals

Synaptic turnover rates, indicated by the number of silver-impregnated lysosomal accumulations, were neither in the dentate gyrus nor in CA3 or CA1 affected by the rearing conditions of social and physical deprivation or enrichment alone. Although there were significant main effects of rearing in the dentate gyrus (ANOVA, rearing *F*_1,21_ = 9.9, *P* < .01) and CA3 (ANOVA, rearing *F*_1,84_ = 10.4, *P* < .01), Post hoc tests did not reveal any significant differences between the two rearing groups (Table 1) and also the pattern of distribution of turnover processes between the layers appeared to be similar (Figures 3(a)–3(d)).

### 3.3. Leveling of Layer-Specific Differences after Adult Wheel Running

Wheel running exerted strong effects on synaptic turnover rates in the dentate gyrus (ANOVA, wheel running, *F*_1,21_ = 50.1, *P* < .0001), CA3 (ANOVA, wheel running *F*_1,84_ = 21.2, *P* < .0001; interaction effect wheel running* layer *F*_3,84_ = 14.7, *P* < .0001), and CA1 (*F*_1,84_ = 54.6, *P* < .0001; wheel running* layer *F*_3,84_ = 6.9, *P* < .001). 

It obviously leveled the foreseen differences in synaptic turnover rates between the layers in both enriched- and deprived-reared individuals mainly by decreasing turnover rates in all layers except the granule or pyramidal cell layer (Figures 3(a)–3(d)). Surprisingly, this decrease was especially significant in CA1, in the stratum radiatum of enriched-reared (Post hoc Unequal N HSD, *P* < .05) and the stratum lacunosum-moleculare of deprived-reared individuals (Post hoc Unequal N HSD, *P* < .05).

### 3.4. No Effects Beyond the Dentate Gyrus and Hippocampus Proper

No effects of either treatment were found beyond the dentate gyrus and hippocampus proper in the entorhinal cortex or subiculum (Table 1, Figures 3(e) and 3(f)).

## 4. Discussion

We quantified naturally occurring presynaptic turnover rates in the hippocampal formation. Synaptic remodeling is a *nondegenerative*, naturally occurring process of structural plasticity in the juvenile and adult brain that occurs in two major forms: structural modification and synapse elimination. Within the synapse, this process is accompanied by autophagy and lysosomal degradation of presynaptic components. Wolff, based on extensive electron microscopic evidence, described this autophagic synapse remodeling as a process by which the efficacy of the synaptic transmission may be reduced or abolished without necessarily a simultaneous disconnection of the synaptic junction [8]. Through the method of silver impregnation of lysosomes by Gallyas et al. [36] the transient accumulations of lysosomes can be visualised and quantified. This has been confirmed by electron microscopy and ultrathin section analyses of silver-stained brain sections in a number of studies [8, 38, 39]. Even to date, Gallyas silver impregnation seems to be the best method to specifically detect synaptic remodeling by staining the transient phase of mainly presynaptic lysosomal accumulations.

We specifically investigated if being deprived of rich social and physical stimuli during the adolescent phase of brain development (pd 21–74) leads to alterations in synaptic turnover rates in young adult animals. This was also evaluated in individuals given an extra four-day exposure to running wheels. Significant layer-specific differences in the level of synaptic turnover were found both in the dentate gyrus and in the CA3 and CA1 regions of the hippocampus proper of our enriched-reared control animals (Figures 3(a)–3(d)). This may be regarded as the typical pattern for healthy young animals. The same pattern of a layer-specific distribution was seen in socially and physically deprived-reared animals. Thus, unlike the finding of much stronger effects in gerbils [5, 16], social and physical deprivation during rearing seemed to have less effect on synaptic remodeling in CD1 mice. However, four days of subsequent wheel running exerted strong effects, both in enriched- and deprived-reared individuals. It led to a leveling of layer-specific differences. This was mainly by decreasing synaptic turnover rates in all layers except for the granule cell and pyramidal cell layers. These effects were limited to the dentate gyrus and hippocampus proper. No effects of our treatment conditions were found for the entorhinal cortex and subiculum. 

In the following we discuss three main questions. We will first address the issue of what might determine the layer-specific differences found in enriched-reared individuals. We consider this experimental group to exhibit rather natural, intact plastic capacities within the hippocampal formation. 

Secondly, we will examine the question of why social and physical deprivation during rearing did not affect synaptic plastic capacities in CD1 mice, such as found in gerbils. Finally, we try to explain how wheel running might affect synaptic turnover in both enriched- and deprived-reared individuals and what the potential relationship might be between the stimulation of cell proliferation and the leveling of layer-specific differences of synaptic turnover rates.

### 4.1. Synaptic Remodeling in the Hippocampal Formation of Enriched-Reared CD1 Mice

The dentate gyrus as well as the CA3 and CA1 region of the hippocampus proper displayed high rates of synaptic turnover, indicating a high dynamic in these structures. Thereby, the acellular layers display the highest and the cellular layers the lowest amount of synaptic remodeling. In the dentate gyrus, high synaptic remodeling rates are conceivable, since especially the inner molecular layer shows high plastic capacities. Following lesions of the perforant path that terminates in the outer molecular layer, commissural and associative fibers normally terminating as recurrents in the inner molecular layer are able to sprout and make contacts with targets in the outer molecular layer, a phenomenon which is not seen *vice versa* (reactive synaptogenesis; [40–42]). Beside the recurrent commissural and associative fibers, interneurons of the subgranular layer innervate proximal dendrites of granule cells [43, 44], all together forming a highly plastic canonical microcircuit [9]. Additional inputs from the septum are also open to shaping or tuning processes (reviewed in [45]). These plastic processes in the inner molecular layer are susceptible to environmental manipulations (naturally occurring synaptic remodeling; [5, 16, 35]). 

The subgranular layer showed similar if not higher levels of synaptic turnover, especially in the infrapyramidal blade. In the subgranular layer, granule cells form synapses with mossy cells and various interneurons ([46]; for details see [47]). In addition, the subgranular layer receives inputs from the septum and dopaminergic, serotoninergic, and noradrenergic afferences(reviewed in [48]). As the only layer of the dentate gyrus, the subgranular layer receives collaterals of especially proximally located CA3 pyramidal cells [47]. Thus, the inner molecular layer and the subgranular layers are similar in that they are the target of feed-back and feed-forward loops and modulatory transmitter influences. 

Furthermore, adult neurogenesis adds structural plasticity to the dentate gyrus, since thousands of new neurons are added to the granule cell layer every day and demand synaptic integration (for reviews see [9, 19, 20]). The inner molecular and the subgranular layer are the first layers in which dendrites (inner molecular) and axons (subgranular layer) of newborn neurons grow, express neurotrophins [49, 50], and demand integration [23, 50, 51]. In this, they first receive GABAergic innervation, which is later partially replaced by glutamate [52]. Both features, the specific connectivity and the challenge to integrate new neurons, may account for the high rates of naturally occurring synaptic turnover in these layers.

A similar layer-specific distribution of synaptic turnover can be seen in CA3 and CA1. As in the dentate gyrus, the acellular layers with commissural, associative and collateral fibers, as well as interneuronal modulation targets, show the highest level of synaptic turnover. This is the stratum radiatum of CA3, in which commissural and associative fibers and inputs from the septum, terminate, and interneurons synapse on the pyramidal dendrites [46, 47]. The stratum radiatum of CA1 too is the target of collaterals (Schaffer collaterals) and commissural projections, septal inputs and interneuronal modulation [46, 47]. This innervation pattern is similar to that of the stratum oriens in CA1, which receives as intensive Schaffer collateral innervation as the stratum radiatum, and thus could explain the rather identical synaptic turnover rates. Interestingly, in the stratum lacunosum-moleculare of CA1 even higher synaptic turnover rates occurred than in the stratum radiatum and stratum oriens of this region. The stratum lacunosum-moleculare is the target of perforant path fibers originating mainly from layer III of the entorhinal cortex (and to a lesser extent from layer II) and it also receives modulatory inputs from the locus coeruleus [53], perirhinal cortex, and thalamus [47]. Perhaps the modulatory influence in particular might account for the high naturally occurring structural plasticity of this layer. 

Astonishingly, the mossy fiber targets in the stratum lucidum in CA3 were not the site of the highest level of synaptic turnover. The extent of the mossy fiber targets is known to be correlated with spatial learning abilities in rats and mice [54, 55]. Furthermore, in rats, an effect of learning on the extent of mossy fiber targets in the stratum lucidum has been shown [56]. However, in mice, mossy fiber sprouting has not been found, potentially indicating a lower plastic potential in mossy fiber targets in this species [57]. This may also explain why in CD1 mice synaptic remodeling in the stratum lucidum was not higher than in the other molecular layers of CA3.

### 4.2. Synaptic Remodeling in Mice Reared under Social and Physical Deprivation

Remarkably, in CD1 mice, no statistically significant effects of social and physical deprivation during rearing could be seen on synaptic remodeling, neither in the dentate gyrus nor in the hippocampus proper, subiculum, or entorhinal cortex. By contrast, in adult gerbils synaptic turnover rates decrease in the inner molecular layer, as well as in the intermediate and outer molecular layer, in response to deprived rearing. In the left hemisphere of gerbils, further decreases were apparent in the subgranular and even in the granule cell layer [16]. However, the gerbil also shows an increase in cell proliferation after deprived rearing [5, 35]. At first sight, this inverse relationship is surprising, since newborn neurons constitute a further source of structural plasticity and one would expect that more newborn neurons should lead to more synaptic remodeling. In fact, it seems that an exhaustion of available synapses is the consequence if too many newborn neurons demand integration, making the inverse relationship between neurogenesis and synaptic remodeling feasible [5, 9, 16]. First data suggest that CD1 mice do not respond to our deprived-rearing paradigm with effects on cell proliferation (in prep.). This might constitute a reason for the missing effect of deprived rearing also on synaptic remodeling in these animals.

### 4.3. Synaptic Remodeling after Wheel Running

Four days of wheel running in adulthood exerted strong effects on synaptic remodeling in the dentate gyrus and hippocampus proper of both socially and physically deprived- and enriched-reared CD1 mice. It led to a leveling of the layer-specific differences mainly by decreasing synaptic turnover rates in all layers except for the granule and pyramidal cell layers. A comparative analysis of wheel running activity during a 4-day wheel running challenge already revealed that CD1 mice reared under enriched or deprived conditions run the same distance and make the same number of running bouts [58]. However, the effects of wheel running did not extend to the subiculum or entorhinal cortex, indicating a lower dynamic and sensitivity to external influences in these regions.

Interestingly, recent data for neurogenesis in CD1 mice showed an enhancement of cell proliferation only in socially and physically deprived-reared CD1 mice (in prep.), while we here found a strong effect of wheel running on synaptic turnover rates in both rearing groups. Hence, factors other than neurogenesis had to play a role in affecting synaptic turnover rates after the wheel running challenge. In fact, wheel running seems to enhance long-term potentiation [31], dendritic growth, complexity, as well as spine densities [32, 33] although not all studies have confirmed this [27, 59]. These latter postsynaptic processes can be directly influenced by, for example, neurotrophic factors like brain-derived neurotrophic factor [60–62], levels of which are enhanced in the dentate gyrus and hippocampus proper in response to wheel running [63–65]. Thus, neurotrophic factors might also affect presynaptic remodeling processes.

The assumption that other, perhaps, intrinsic influences must account for the effects of wheel running on synaptic remodeling is supported by the fact that altered synaptic turnover rates did not only occur in the dentate gyrus, but also in CA3 and CA1. Axons of newborn neurons reach CA3 already 4–10 days after birth [59, 66], thus in the time window in which we analyzed synaptic turnover after wheel running. However, if alterations in the number of newborn neurons already affected synaptic plasticity in the hippocampus proper, one would expect the greatest effects immediately downstream of the dentate gyrus in the mossy fiber termination field, the stratum lucidum of CA3, and not in CA1. It seems, in fact, that in CD1 mice CA3 and CA1 also exhibit intrinsic plasticity. This assumption finds support in recent findings of a systematic analysis of synaptic turnover rates in diverse cortical areas of the gerbil, showing that associative cortical areas also display synaptic remodeling, and region—as well as layer-specific differences which appear mainly determined by local and distant associative connections [7]. These layer-specific differences were leveled after environmental deprivation during rearing, an effect that parallels our findings after wheel running in CD1 mice.

## Figures and Tables

**Table 1:** Absolute numbers of silver-stained precipitates in equally sized measuring fields in different hippocampal regions and laminae.

			Number of precipitates									
Measuring field			ER	DR			ER after wheel running			DR after wheel running		
			Value (abs)	Value (abs)	difference ER − DR (%)	*P*	value (abs)	difference ER − ER after wheel running (%)	*P*	Value (abs)	difference DR − DR after wheel running (%)	*P*
dentate gyrus	oML	spb	224	242	8	n.s.	195	−13	n.s.	181	−25	n.s.
		ipb	217	216	−1	n.s.	167	−23	n.s.	194	−10	n.s.
	mML	spb	224	240	7	n.s.	182	−19	n.s.	182	−24	n.s.
		ipb	233	240	3	n.s.	168	−28	n.s.	184	−23	n.s.
	iML	spb	270	291	8	n.s.	205	−24	n.s.	228	−21	n.s.
		ipb	258	275	7	n.s.	190	−26	n.s.	209	−24	n.s.
	gcl	spb	92	106	16	n.s.	70	−23	n.s.	125	18	n.s.
		ipb	109	131	20	n.s.	76	−31	n.s.	129	−2	n.s.
	sgl	spb	292	334	14	n.s.	228	−22	n.s.	265	−21	n.s.
		ipb	344	381	11	n.s.	250	−27	n.s.	284	−25	n.s.

CA3	So		230	265	15	n.s.	164	−29	n.s.	171	−35	n.s.
	Sp		91	105	16	n.s.	127	40	n.s.	224	113	.09
	Sl		267	292	9	n.s.	159	−41	.09	208	−29	n.s.
	Sr		333	368	11	n.s.	221	−34	.07	253	−31	n.s.
CA1	So		273	284	4	n.s.	184	−33	n.s.	169	−40	.06
	Sp		57	89	56	n.s.	62	8	n.s.	94	6	n.s.
	Sr		294	302	3	n.s.	179	−39	**<.05**	195	−36	n.s.
	Slm		335	378	13	n.s.	250	−25	n.s.	249	−34	**<.05**
EC	L II		213	170	−20	n.s.	201	−6	n.s.	202	19	n.s.
	L III		269	219	−18	n.s.	195	−28	n.s.	196	−11	n.s.

Subiculum			250	267	7	n.s.	224	−11	n.s.	237	−11	n.s.

ER: reared under socially and physically enriched conditions (pd 21–74); DR: reared under socially and physically deprived conditions; after wheel running: after an additional wheel running challenge in adulthood (pd 74–78); oML: outer molecular layer; mML: intermediate molecular layer; iML: inner molecular layer; gcl: granule cell layer; sgl: subgranular layer; spb: suprapyramidal blade; ipb: infrapyramidal blade; So: stratum oriens, Sp: stratum pyramidale; Sl: stratum lucidum; Sr: stratum radiatum; Slm: stratum lacunosum-moleculare; EC: entorhinal cortex; L: lamina; statistical differences between laminae only shown in Figure 3.
